# Impact of early preeclampsia prediction on medication adherence and behavior change: a survey of pregnant and recently-delivered individuals

**DOI:** 10.1186/s12884-024-06397-z

**Published:** 2024-03-13

**Authors:** Alison Cowan, Carrie Haverty, Reece MacDonald, Arkady Khodursky

**Affiliations:** Mirvie, Inc., 820 Dubuque Ave, South San Francisco, CA 94080 USA

**Keywords:** Preeclampsia, Pregnancy, Predictive testing, Biomarkers, Medication adherence, Medication hesitancy

## Abstract

**Background:**

Behavior change and medication adherence represent potential barriers to optimal prevention of pregnancy complications including preeclampsia. We sought to evaluate baseline sentiments on pregnancy care and medication amenability, and how these measures would be impacted by early predictive testing for preeclampsia.

**Methods:**

We developed a digital survey to query participants’ baseline sentiments on pregnancy care, knowledge about pregnancy complications, and views on a hypothetical test to predict preeclampsia. The survey was administered online to pregnant and recently-delivered individuals in the United States. Survey data were analyzed using pooled two-sample proportion z-tests with adjustment for multiple comparisons.

**Results:**

One thousand and twenty-two people completed the survey. 84% reported they were satisfied with their pregnancy care. Self-assessed knowledge about preeclampsia was high, with 75% of respondents reporting they have a “good understanding” of preeclampsia, but measured knowledge was low, with only 10% able to identify five common signs/symptoms of preeclampsia. Notably, 40% of participants with prior preeclampsia believed they were at average or below-average risk for recurrence. 91% of participants desired early pregnancy predictive testing for preeclampsia. If found to be at high risk for preeclampsia, 88% reported they would be more motivated to follow their provider’s medication recommendations and 94% reported they would desire home blood pressure monitoring. Increased motivation to follow clinicians’ medication and monitoring recommendations was observed across the full spectrum of medication amenability. Individuals who are more medication-hesitant still reported high rates of motivation to change behavior and adhere to medication recommendations if predictive testing showed a high risk of preeclampsia. Importantly, a high proportion of medication-hesitant individuals reported that if a predictive test demonstrated they were at high risk of preeclampsia, they would feel more motivated to take medications (83.0%) and aspirin (75.9%) if recommended.

**Conclusion:**

While satisfaction with care is high, participants desire more information about their pregnancy health, would value predictive testing for preeclampsia, and report they would act on this information. Improved detection of at-risk individuals through objective testing combined with increased adherence to their recommended care plan may be an important step to remedy the growing gap in prevention.

**Supplementary Information:**

The online version contains supplementary material available at 10.1186/s12884-024-06397-z.

## Background

Pregnancy care in the U.S. is evolving, as evidenced by a rise in telehealth, remote monitoring, and ACOG’s Redesigning Prenatal Care Initiative [1, 2]. In addition, new technologies are emerging that will enable the objective early prediction of pregnancy complications such as preeclampsia [3–7] to facilitate intervention. Preeclampsia, a disorder of high blood pressure in pregnancy that can cause organ dysfunction and preterm birth, is a leading cause of maternal and neonatal morbidity and mortality in the U.S. and globally, making it a critical target for improving maternal-child health [8]. Guidance regarding the optimal preventive care plan for individuals at high risk for preeclampsia became available in 2023 and can be applied to those identified to be at risk [8].

This evidence-based guidance recommends interventions including remote monitoring, medications, and lifestyle and behavioral interventions to optimize the prevention of preeclampsia [8]. However, patient adherence to recommendations that require behavioral change and medication uptake has historically been challenging [9–11], and attempted educational interventions have shown mixed results [12]. However, evidence from other fields suggests that objective risk stratification can be a strong motivator for behavior change, as in the case of coronary artery calcium scoring and its association with significantly improved statin adherence and weight loss [13–17]. Therefore, we sought to understand how this could translate to an obstetrical population, and whether having a test to objectively predict the risk of preeclampsia in advance of symptoms would drive behavior change and medication adherence. Given that preeclampsia affects an estimated 8% of pregnancies in the U.S. and has been on the rise [18], improved medication adherence and behavior change to prevent preeclampsia could drive substantial public health benefit if successful.

As predictive testing for preeclampsia in an asymptomatic population has not been available in the U.S., there is scant evidence regarding how patients would respond to such testing. This study addresses this gap by evaluating attitudes toward predictive testing for pregnancy complications such as preeclampsia in pregnant and recently-delivered individuals and their anticipated behavior change resulting from such testing.

## Methods

An online English-language questionnaire was developed building on a prior study [19] of individuals with a history of preeclampsia that was developed in collaboration with the Preeclampsia Foundation, clinicians, and social scientists. The present study built on this prior work related to patient values and preferences, and was developed primarily by A. Cowan, an OB/GYN, and C. Haverty, a genetic counselor, with input on wording and clarity from a third-party professional polling entity. The survey included demographic and clinical history questions, as well as questions related to the pregnancy experience and degree of satisfaction (9 questions), patient self-assessed and measured knowledge about preeclampsia and other pregnancy complications (12 questions), views on preeclampsia and access to information about it (7 questions), and questions on how they would view a test to predict preeclampsia (8 questions.) This convenience survey was fielded by an independent company specializing in the creation of nationally-representative surveys, with a 64% completion rate. The third-party polling agency works with panel partners who recruit and supply the sample for research, maintaining a sample of potential respondents who have opted in to participate in this type of research. Panel members were sent invitations to participate in research with a variety of delivery methods (e.g., email, text, in-app alerts) with the goal of bringing in respondents with a diversity of motivation to participate.

Both positively- and negatively-framed questions were used to minimize bias. A variety of question types were used, including some knowledge-based questions and some Likert scale questions. The question order remained the same for each respondent, however for appropriate questions the response items were randomized to prevent order bias. The distribution of scores was collapsed into binary “agree” vs. “disagree” categories, except where otherwise noted. The full questionnaire is available in the Appendix. Only complete questionnaires were recorded.

The survey included a total of 36 questions and used adaptive questioning (e.g., individuals reporting they were unfamiliar with preeclampsia were not asked further questions about their level of knowledge on preeclampsia or its signs/symptoms.) Responses were collected digitally using digital fingerprinting to prevent the same computer accessing the survey more than once. In addition, timestamp/length of interview was included as part of the third party polling agency’s standard quality/fraud prevention checks and was reviewed for all respondents.

Participant responses were categorized as “medication hesitant” versus “medication amenable” at baseline, in response to the question, “How likely would you be to take medications, such as aspirin, throughout your pregnancy if your healthcare provider(s) recommended it?” Respondents who answered they were “somewhat likely” or “very likely” were classified as medication amenable, and those who answered that they were “somewhat unlikely” or “very unlikely” were classified as medication hesitant. Participant responses to additional questions were stratified by their baseline amenability to medications to better understand how behavior change might be different between these two groups.

Prior to answering questions related to preeclampsia prediction, respondents were provided context in lay terms surrounding predictive testing for preeclampsia (see Appendix for full text.) A definition of preeclampsia and background including that it impacts an estimated 8% of pregnancies [18] was provided. They were also told that there is currently no reliable way to predict preeclampsia outside of clinical risk factors, which perform suboptimally. Respondents were informed that some interventions such as aspirin and blood pressure monitoring can prevent or delay the onset of symptoms in individuals at high risk. After reading this information about preeclampsia, respondents were asked a series of questions about a hypothetical blood test to predict preeclampsia.

Data collection was carried out via online recruitment. Participants were eligible if they reported being currently pregnant or delivered within the last year and were U.S. residents ages 18–45, with the exception of Alabama, Nebraska, and Mississippi. Residents of these states were required to be at least 19 (AL and NE) and 21 (MS) in accordance with state law. Participants were recruited from online consumer panels using general population samples in the United States, and completed a short qualification screening survey. In order to achieve a diverse sample, we aimed to survey at least 100 Asian respondents, 200 Black respondents, 250 Hispanic respondents, and 400 White respondents, with at least 50 not identifying with the four preceding categories. Race and ethnicity were recorded by self-report. All reporting of race and ethnicity in this study is in individuals who reported being a single race and only among Asian, Black, Hispanic, and White individuals, due to insufficient numbers for other races to allow for meaningful analysis. After completing the qualification survey, informed consent was obtained to continue participation in the web-based survey, which was deemed exempt by the institutional review board. 1,022 individuals completed the survey in September 2022.

Data were weighted by race and where necessary by education, age, region, income, size of household, marital status, and for propensity to be online to bring them in line with their actual proportions in the population using publicly available targets from the March 2021 Current Population Survey (CPS.) Propensity scoring was used to minimize potential bias associated with Internet-based panel samples. The consistency of responses within each questionnaire category was confirmed by establishing that Cronbach’s alpha was greater than 0.7. For each attitude question in the questionnaire, we compared proportions of “Agree” answers, after converting Likert-scaled responses to a binarized “Agree”/”Disagree”, between all pairs of specified groups of respondents. We used Z-statistic to assess whether the observed difference in proportions was significant, assuming the null hypothesis that there was no difference between the groups. Whenever applicable we controlled for the False Discovery Rate across all comparisons by using the Benjamini-Hochberg (BH) procedure. The effect size was evaluated by sample standardized differences and corresponding 95% confidence intervals. Statistical analysis was performed in R 4.3.1 (R Core Team. (2022). R: A language and environment for statistical computing (Version 4.3.1). R Foundation for Statistical Computing, Vienna, Austria. URL: https://www.R-project.org/).

## Results

Baseline characteristics of the population studied are reported in Table 1. A total of 793 individuals (77.6%) reported being medication amenable at baseline, and 229 (22.4%) were medication hesitant. There was no difference in medication hesitancy across age groups, pregnancy status (whether currently pregnant or recently delivered), or gravidity. White participants were significantly more likely than Black and Hispanic participants to be medication amenable (82.1% vs 70.2% and 74.4% respectively). Medication amenability was also positively associated with increasing educational levels and income. Relationship status was associated with medication amenability, with married/partnered participants more likely to be medication amenable (80.7% of married/partnered vs. 70.6% of single participants). Finally, a history of prior preeclampsia or preterm birth was not associated with medication amenability, but a history of gestational diabetes was associated with a higher degree of medication amenability compared to individuals without a history of pregnancy complications (88.7% vs. 77.4%).

**Table 1:** Baseline characteristics, medication-hesitant versus -amenable

Category	Medication Amenable N (%)	Medication Hesitant N (%)	Total (N, %)	*P*-value*	95% CI	Effect size, d
Age				all NS		
18–24	124 (76.1)	39 (23.9)	163 (15.9)			
25–34	420 (76.8)	127 (23.2)	547 (53.5)			
35–45	250 (80.1)	62 (19.9)	312 (30.5)			
Race and ethnicity						
Asian	42 (80.8)	10 (19.2)	52 (5.2)			
Black	113 (70.2)	48 (29.8)	161 (16.1)	0.01^B^^−^^W^	(-0.2, -0.04)	-0.283
Hispanic	201 (74.4)	69 (25.6)	270 (27.1)	0.04^H^^−^^W^	(-0.14, -0.01)	-0.187
White	423 (82.1)	92 (17.9)	515 (51.6)			
Pregnancy Status^§^				all NS		
Currently pregnant	307 (66.4)	82 (21.1)	389 (38.1)			
Recently delivered	500 (76.8)	151 (23.2)	651 (63.7)			
Income						
< $50,000 (l)	150 (66.4)	76 (33.6)	226 (22.1)			
$50,000-$99,999 (m)	225 (78.1)	63 (21.9)	288 (28.2)	< 0.001^ m−^^l^	(0.04, 0.2)	0.265
$100,000 or more (h)	419 (82.3)	90 (17.7)	509 (49.8)	< 0.001^ h−l^	(0.09, 0.23)	0.371
Education						
High school or less (I)	130 (68.4)	60 (31.6)	190 (9.4)	< 0.001^I^^−^^III^	(-0.23, -0.07)	-0.351
Vocational/Assoc (II)	296 (75.7)	95 (24.3)	391 (38.3)	0.01^II^^−^^III^	(-0.13, -0.02)	-0.187
4-year college degree or more (III)	367 (83.2)	74 (16.8)	441 (43.2)			
Marital status						
Married/Partnered	572 (80.7)	137 (19.3)	709 (69.4)	< 0.001	(0.04, 0.16)	0.236
Single	221 (70.6)	92 (29.4)	313 (30.6)			
Pregnancy complications						
Preeclampsia	131 (80.4)	32 (19.6)	163 (15.9)			
Preterm birth	147 (78.6)	40 (21.4)	187 (18.3)			
Gestational diabetes	125 (88.7)	16 (11.3)	141 (13.8)	0.041**	(0.05, 0.18)	0.303
None	483 (77.4)	141 (22.6)	624 (61.1)			
Decline to answer	16 (66.7)	8 (33.3)	24 (2.3)			

^*^ Only significant *P*-values after adjusting for multiple comparisons (FDR < 0.05) are reported

^**^ Gestational diabetes compared to no complications

^B^^−^^W^ Black respondents compared to White respondents

^H^^−^^W^ Hispanic respondents compared to White respondents

^§^ Some individuals reported both being currently pregnant and delivered in the last 12 months

^m^^−^^l, h^^–^^l^ Individuals making both $50,000-$99,999 and $100,000 or more were more medication amenable than individuals making < $50,000

^I^^−^^III^ High-school or less compared to 4-year college degree or more

^II^^−^^III^ Vocational/associate compared to 4-year college degree or more

### Satisfaction with care, sentiments on pregnancy care, personalized care, and feeling heard

Most individuals reported high satisfaction with their pregnancy care overall, with 84.2% (*N* = 861) reporting they were somewhat or very satisfied with their care. Most respondents felt that having more information about their pregnancy would give them more control over their care choices, enable better conversations with their healthcare providers, and enable them to feel more empowered (Table 2). Overall, participants expressed a high desire for personalized pregnancy care (93.9%), high trust in their healthcare provider (91.7%), and reported that they would desire an early test to predict their risk of pregnancy complications if one were available (91.0%). There were no statistically significant differences by race or ethnicity observed in the responses related to satisfaction with care (Supplemental Table 1) and pregnancy sentiments on care, personalized care, and feeling heard were similar. White respondents were more likely than Hispanic respondents to report that the more information they have about their pregnancy care, the more empowered they feel (*P*-value = 0.001, proportion difference 95% CI = (0.03, 0.11), effect size = 0.273). There were no other differences observed by race or ethnicity in evaluating pregnancy sentiments on care, personalized care, or feeling heard (Supplemental Table 2).

**Table 2:** Participant sentiments on pregnancy care, personalized care, and feeling heard: medication-amenable vs. -hesitant individuals (Cronbach’s alpha = 0.76)

Statement	Total N (%)	Medication amenable N (%)	Medication hesitant N (%)	*P*-val	Prop diff: 95% CI	Effect size, (d)	d: 95% CI
The more information I have about my pregnancy, the more control I have over my care choices.	983 (96.2)	770 (97.1)	213 (93.4)	0.01	(0.003, 0.071)	0.175	(0.028, 0.322)
The more information I have about my pregnancy, the better the conversations are with my healthcare providers(s).	976 (95.4)	766 (96.5)	211 (92.1)	0.005	(0.006, 0.08)	0.191	(0.044, 0.338)
The more information I have about my pregnancy, the more empowered I feel.	962 (94.1)	758 (95.6)	204 (89.1)	< 0.001	(0.022, 0.108)	0.246	(0.099, 0.393)
I would like my prenatal care to be personalized to my individual pregnancy.	960 (93.9)	760 (95.8)	200 (87.3)	< 0.001	(0.04, 0.13)	0.309	(0.161, 0.457)
I trust the healthcare provider(s) who manage my pregnancy (such as an OB/GYN or midwife).	937 (91.7)	738 (92.9)	200 (87.3)	0.007	(0.009, 0.103)	0.188	(0.041, 0.335)
If there were a test that could predict my risk of complications in pregnancy, I would want it.	930 (91.0)	730 (91.9)	200 (87.3)	0.033	(-0.001, 0.093)	0.151	(0.004, 0.298)
Overall, I feel my prenatal care has been personalized to meet my needs.	889 (87.0)	701 (88.4)	188 (82.1)	0.013	(0.009, 0.117)	0.178	(0.031, 0.325)
I feel heard when bringing up concerns about symptoms or concerns related to possible pregnancy complications with my healthcare provider.	876 (85.7)	689 (86.9)	187 (81.7)	0.047	(-0.003, 0.108)	0.143	(-0.004, 0.29)
I have felt at times like I don’t understand certain aspects of my pregnancy care as well as I would like to.	598 (58.5)	460 (58.0)	138 (60.5)	0.496	(-0.097, 0.047)	-0.051	(-0.198, 0.096)

There were notable differences in these measures between medication hesitant and amenable individuals. Satisfaction with care was high overall for both groups, however medication hesitant respondents were less likely to be satisfied with their care compared to medication amenable respondents (77.3 vs. 86.3%, *P*-value = 0.001, 95% CI = (-0.152, -0.027), effect size = -0.234). Medication hesitant individuals were also less likely to report that more information would give them more control over their choices (93.4% vs. 97.1%, *P*-value = 0.017, 95% CI = (-0.071, -0.003), effect size = -0.175), enable better conversations with healthcare providers (92.1% vs 96.5%, *P*-value = 0.009, 95% CI = (-0.08, -0.006)), effect size = -0.191, make them feel more empowered (89.1% vs 95.6%, *P*-value < 0.001, 95% CI = (-0.108, -0.022,), effect size = -0.246), want to receive more personalized care (87.3% vs 95.8%, *P*-value < 0.001, 95% CI = (-0.13, -0.04), effect size = -0.309) and to trust their healthcare provider (87.3% vs 92.9%, *P*-value = 0.01, 95% CI = (-0.103, -0.009), effect size = -0.188) They were also less likely, albeit less precisely, to desire a test to predict preeclampsia (87.3% vs 91.9%, *P*-value = 0.045, 95% CI = (-0.093, 0.001), effect size =—0.151 ± 0.147) and less likely to feel their care has been personalized to meet their needs (82.1% vs 88.4%, *P*-value = 0.017, 95% CI = (-0.117, -0.009), effect size = -0.178) though overall the majority of individuals in both groups responded favorably to all of the preceding statements (Table 2).

### Self-reported knowledge about pregnancy complications

Respondents were asked about their self-assessed knowledge of gestational diabetes, Down syndrome, and preeclampsia. Patient self-reported knowledge was similar across these conditions, at 59.4, 51.2, and 50.3% of respondents reporting they are “extremely” or “very knowledgeable” about these conditions respectively. Compared with individuals without a history of preeclampsia, individuals with a history of preeclampsia reported significantly higher levels of knowledge on gestational diabetes (74 vs. 57%, *P*-value < 0.001, 95% CI = (0.097, 0.254), effect size = 0.375), preeclampsia (81 vs. 44%, *P*-value < 0.001, 95% CI = (0.293, 0.438), effect size = 0.815) and Down syndrome (60 vs. 49%, *P*-value = 0.02, 95% CI = (0.02, 0.192), effect size = 0.215). There were no differences in self-reported knowledge of pregnancy complications between medication amenable vs. hesitant individuals.

To better understand self-reported knowledge, participants were also asked how they obtain pregnancy information and their level of confidence in doing so (Table 3). The majority of respondents had high confidence in their ability to obtain pregnancy information and that they understood their risk of preeclampsia well, yet the majority also reported a desire for more information about their risk of preeclampsia and other pregnancy complications. Despite their similar self-reported knowledge about pregnancy complications, medication hesitant individuals were less likely to feel confident in their ability to obtain answers about preeclampsia and pregnancy complications (81.6 vs 89.4%, *P*-value = 0.002, 95% CI = (-0.133, -0.024), effect size = -0.223), to feel that they had a good understanding of preeclampsia (65.4 vs. 78.4%, *P*-value < 0.001, 95% CI = (-0.199, -0.063), effect size = -0.292), and to feel fully informed about their personal risk of complications in pregnancy (68.1 vs. 76.4%, *P*-value = 0.011, 95% CI = (-0.15, -0.016), effect size = -0.186) (Table 3).

**Table 3:** Self-reported knowledge of preeclampsia, confidence, and desire for more knowledge: medication-amenable vs. -hesitant individuals (Cronbach’s alpha = 0.83)

Statement	Total	Medication amenable, N (%)	Medication hesitant, N (%)	*P*-val	Prop diff: 95% CI	Effect size, (d)	d: 95% CI
I feel confident in my ability to get answers about preeclampsia and other pregnancy complications.	895 (87.6)	709 (89.4)	186 (81.6)	0.002	(0.024, 0.133)	0.223	(0.075, 0.371)
Outside of my healthcare provider(s), I know where to go to get reliable information about preeclampsia and other pregnancy complications.	787 (77.0)	605 (76.2)	182 (79.5)	0.299	(-0.093, 0.027)	-0.08	(-0.227, 0.067)
I feel I have a good understanding of preeclampsia.	772 (75.5)	622 (78.4)	149 (65.4)	< 0.001	(0.063, 0.199)	0.292	(0.144, 0.44)
I feel fully informed about my personal risk of complications during pregnancy, including preeclampsia.	763 (74.7)	607 (76.4)	156 (68.1)	0.011	(0.016, 0.15)	0.186	(0.039, 0.333)
I feel I understand my personal risk of preeclampsia well.	757 (74.1)	596 (75.2)	160 (70.2)	0.130	(-0.017, 0.116)	0.112	(-0.035, 0.259)
I wish I knew more about my risk of preeclampsia and other pregnancy complications.	683 (66.8)	527 (66.5)	156 (68.4)	0.579	(-0.088, 0.049)	-0.041	(-0.188, 0.106)
Before getting pregnant, I understood my risk of preeclampsia and other pregnancy complications.	570 (55.8)	441 (55.6)	129 (56.3)	0.847	(-0.08, 0.066)	-0.014	(-0.161, 0.133)

### Assessed knowledge about pregnancy complications

Following self-assessed knowledge on pregnancy complications, objective assessment of knowledge revealed that many individuals had overestimated their knowledge base. Among the 982 individuals who indicated at least some familiarity with preeclampsia at baseline, 90.8% could identify at least one sign/symptom of preeclampsia, however, only 9.6% could identify the 5 common signs/symptoms (described in the questionnaire as elevated blood pressure, swelling, headache, visual changes, and stomach pain.) Those who reported being extremely/very knowledgeable about preeclampsia were more likely to correctly identify at least one correct warning sign/symptom (97% vs 84%, *P*-value < 0.001, 95% CI = (0.097, 0173), effect size = 0.474) and were more likely to identify all 5 signs/symptoms (14% vs 4%, *P*-value < 0.001, 95% CI = (0.066, 0.141), effect size = 0.359). Participants who had previously experienced preeclampsia were also more likely to identify all 5 signs/symptoms, but the majority were still unable to do so (18% vs 8%, *P*-value < 0.001, 95% CI = (0.04, 0.172), effect size = 0.318). When asked to assess their risk of developing preeclampsia in a future pregnancy, 34% of respondents with a history of preeclampsia felt they were at average risk, and 6% believed themselves to be at low risk of developing preeclampsia.

Measured knowledge of preeclampsia was similar between medication hesitant vs. amenable individuals; although medication hesitant individuals were somewhat less likely to be able to identify one sign/symptom of preeclampsia, most were able to do so (87.1 vs. 91.9%, *P*-value = 0.04, 95% CI = (-0.098, 0.005), effect size = -0.153 ± 0.151).

In total, 90% of participants were not able to identify all of the common preeclampsia warning signs/symptoms, despite 75% of participants feeling they had a “good understanding” of preeclampsia overall.

### Desire for early predictive testing for pregnancy complications and anticipated behavior change

Regarding a hypothetical blood test to predict preeclampsia, the majority of respondents reported a desire for testing and that they would change their behavior if such a test were available (Table 4). Overall, 90.8% reported that they would want to have a predictive test even if that test were not 100% accurate. It is foreseeable that a test to predict preeclampsia could increase patient anxiety, however only 21.9% of respondents reported this to be the case, with 57.6% stating that it would give them peace of mind (irrespective of their test results).

**Table 4:** Blood test for preeclampsia prediction: impact on care and self-reported motivation, medication-amenable vs. -hesitant (Cronbach’s alpha = 0.77)

Statement	Total	Medication amenable, N (%)	Medication hesitant, N (%)	*P*-val	Prop diff: 95% CI	Effect size, (d)	d: 95% CI
If a screening test told me I was at high risk for preeclampsia, I would want to discuss the signs and symptoms of preeclampsia with my healthcare provider.	968 (94.7)	766 (96.6)	202 (88.6)	< 0.001	(0.037, 0.123)	0.309	(0.161, 0.457)
If a screening test told me I was at higher risk for preeclampsia, I would expect that my healthcare provider would make a personalized plan for pregnancy care.	965 (94.4)	763 (96.1)	203 (88.6)	< 0.001	(0.032, 0.118)	0.285	(0.137, 0.433)
If a screening test told me I was at higher risk for preeclampsia, I would be interested in options to monitor my blood pressure at home.	963 (94.2)	752 (94.8)	210 (92.1)	0.120	(-0.011, 0.065)	0.109	(-0.038, 0.256)
If a prediction test showed my risk to develop preeclampsia was low, I would feel more at ease about my prenatal care.	936 (91.6)	742 (93.6)	194 (85.1)	< 0.001	(0.036, 0.134)	0.278	(0.13, 0.426)
Even if a prediction test were not 100% accurate, I would want to take a test early in my pregnancy that lets me know my chances of developing a problem like preeclampsia.	928 (90.8)	734 (92.4)	194 (85.1)	0.001	(0.024, 0.123)	0.233	(0.085, 0.381)
If I better understood the risks of preeclampsia, I would be more motivated to follow my healthcare provider(s) medication recommendations.	898 (87.9)	708 (89.3)	190 (83.0)	0.010	(0.01, 0.116)	0.183	(0.036, 0.33)
If I better understood the risks of preeclampsia, I would be more motivated to follow my health care provider(s) recommendation to take baby aspirin.	890 (87.1)	717 (90.4)	173 (75.9)	< 0.001	(0.086, 0.205)	0.395	(0.247, 0.543)
It would help me feel more confident about notifying my care team with concerns about signs or symptoms.	603 (59.0)	489 (61.7)	114 (49.8)	0.001	(0.046, 0.192)	0.241	(0.094, 0.388)
It would give me some peace of mind.	589 (57.6)	472 (59.5)	117 (51.1)	0.023	(0.011, 0.157)	0.170	(0.023, 0.317)
I would want to set and follow a personalized treatment plan with my care team.	517 (50.6)	441 (55.6)	76 (33.2)	< 0.001	(0.154, 0.294)	0.463	(0.315, 0.611)
It would empower me to advocate for myself during my pregnancy.	473 (46.2)	391 (49.3)	82 (35.8)	< 0.001	(0.064, 0.206)	0.276	(0.128, 0.424)
It would help me feel more engaged with my pregnancy and care team.	423 (41.4)	338 (42.6)	85 (37.1)	0.136	(-0.016, 0.126)	0.113	(-0.034, 0.26)
I would make different choices in the management of my pregnancy.	418 (40.9)	347 (43.8)	71 (31.0)	0.001	(0.058, 0.197)	0.267	(0.12, 0.414)
It would help strengthen my relationship with my care team.	301 (29.5)	254 (32.0)	47 (20.5)	0.001	(0.053, 0.177)	0.264	(0.117, 0.411)
It would add to my anxiety.	224 (21.9)	172 (21.7)	52 (22.7)	0.743	(-0.072, 0.051)	-0.024	(-0.171, 0.123)

If individuals were told that they were at high risk of preeclampsia based on a screening test, 94.7% would want to discuss the signs and symptoms with a provider, 94.4% would desire a personalized care plan for their pregnancy, and 94.2% would be interested in home blood pressure monitoring. Overall, 87.9% would be more motivated to follow their provider’s medication recommendations if they better understood the risks of preeclampsia, with 87.1% reporting that they would be more motivated to take aspirin if recommended. There were no differences observed across race and ethnicity and participants’ reported desire for predictive testing nor in their self-reported anticipated responses to such testing (Supplemental Table 3).

A high proportion of both medication hesitant and medication amenable individuals reported that they would desire a test for preeclampsia prediction and would act on the results, with the majority reporting they would want to discuss the signs and symptoms of preeclampsia with their healthcare provider, make a personalized plan for pregnancy care, and to monitor their blood pressure at home (Table 4). Medication hesitant individuals were slightly less likely to desire the test and take the aforementioned actions compared with medication amenable individuals. However, a high proportion of both medication hesitant and medication amenable individuals reported that if a predictive test demonstrated they were at high risk of preeclampsia, they would feel more motivated to take medications as recommended (83.0 vs. 89.3%, *P*-value = 0.010, 95% CI = (0.01, 0.116), effect size = 0.183 ± 0.147) and to take aspirin if recommended (75.9 vs. 90.4%, *P*-value < 0.001, 95% CI = (0.086, 0.205), effect size = 0.395 ± 0.148) (Table 4).

## Discussion

This large, diverse survey of pregnant and recently-delivered individuals demonstrates that while satisfaction with care is high overall, patients desire more information about their pregnancy and would value objective testing to better understand their risk of preeclampsia. Participants with both high and low baseline medication amenability reported they would be more motivated to follow medication and monitoring recommendations with predictive testing for preeclampsia, indicating that objective risk stratification could meaningfully improve medication adherence and behavior change.

Our findings are consistent with and build upon existing literature in several respects. We found gaps in participant knowledge, consistent with previous research indicating that while most are familiar with preeclampsia, the majority of patients are unable to identify its common signs and symptoms [20, 21]. In addition, although prior preeclampsia is a known risk factor [22, 23], 40% of participants with prior preeclampsia believed they were at average or below-average risk for recurrence. This represents an educational opportunity for those at elevated risk.

Our study also builds on prior work done in individuals with a history of preeclampsia demonstrating that the majority of such individuals would value predictive testing for preeclampsia [19]. Our study confirms this finding in a general obstetrical population.

Medication amenability at baseline and in response to potential predictive testing was of particular interest given low rates of medication adherence previously observed, including during pregnancy [11, 24, 25]. Even with life-threatening conditions, medication non-adherence is prevalent, with almost one in four patients failing to fill a single prescribed medication after hospitalization for acute myocardial infarction [26]. Poor adherence, impacting approximately 50% of non-pregnant [24] and pregnant [11] individuals, has been shown to decrease treatment efficacy and worsen outcomes [24, 27, 28]. As such, it represents a significant area of concern for clinicians, healthcare systems, and payers [25].

Previously, authors have examined reasons for medication non-adherence in pregnancy and ways to improve it. Efforts to improve adherence in pregnancy have yielded mixed results, and there remains limited evidence about the potential impact of available behavioral and educational interventions [12]. Examination of the reasons behind low medication adherence have yielded insights such as the perceived medicalization of pregnancy, not realizing medication had been recommended, and not feeling that the risk applied to them, despite having stratified as high risk based on clinical factors such as race, age, and BMI [29].

This final insight—not feeling that the risk applied to them—ties to the current study. Given the non-obstetrical literature demonstrating that objective risk stratification is associated with positive behavior change and improved medication adherence [13–17], we wanted to understand the potential impact of objective risk prediction for preeclampsia. Baseline medication amenability was observed across a continuum in the present study, and participants across the entire spectrum reported that their behavior would be positively impacted by predictive testing. It appears that having an objective test to predict preeclampsia would motivate improved medication adherence, uptake of remote patient monitoring, and targeted education, which have been recommended in the recent care plan for individuals at high risk of preeclampsia and are associated with prevention of preeclampsia and improved obstetrical outcomes [8, 30]. While we did not assess willingness to undertake additional lifestyle changes, it is possible that having objective risk prediction could improve these as well.

An unexpected finding of the study was that there were no differences observed overall with satisfaction of care nor in feeling heard by race and ethnicity. Interestingly, the high satisfaction with care in our study mirrors that seen in a large contemporary survey of the U.S. population, in which 90.5% of all respondents reported being “very or somewhat” satisfied with their care, without differences observed across race and ethnicity [31]. However, that study asked additional questions which uncovered important differences in experience with care by race and ethnicity: Black, Hispanic, and multiracial patients in fact reported the highest prevalence of mistreatment by a healthcare provider during pregnancy. Notably, 75% of individuals in that survey who reported any pregnancy mistreatment were nevertheless satisfied with their care during pregnancy. Thus, it’s clear that there are crucial measures of the care experience beyond satisfaction that were beyond the scope of the present study.

In addition, Black and Hispanic individuals did have lower medication amenability, which itself was associated with lower satisfaction with care and lower levels of trust in the healthcare provider. A stratified analysis by race across medication hesitancy was not possible in this study due to limited sample sizes in each stratum, and a larger study to explore the relationship between race, ethnicity, and medication amenability would be helpful to better assess these important relationships.

Our study has several strengths, including a large diverse sample size of currently and recently pregnant individuals. There are also limitations. Due to the recruitment method used, it was not possible to calculate response rates, and selection bias cannot be ruled out. The survey had a moderate completion rate of 64%, and as only complete surveys were analyzed, it is not possible to comment on potential underlying differences between those who partially completed the survey versus those who finished it. Individuals who responded to the survey could represent a more satisfied cohort compared to non-responders, and this may have contributed to the high patient satisfaction rates observed in the present study. In addition, this survey was conducted online; thus, the sampled population may be of higher than average literacy levels and reflects a population with Internet access. The survey was also conducted only in English, which limits its generalizability to non-English-speaking populations. Finally, participants’ anticipated behavior change might not be predictive of actual behavior change, and this will be an important area for future study. Future study to prospectively measure behavior change and medication adherence in response to an objective test would be the optimal next step to confirm the present study’s findings.

In sum, this study demonstrates important insights about pregnant individuals’ desire for more pregnancy-related information, and opportunities for improved targeted preeclampsia and pregnancy complication education. If early predictive testing for preeclampsia and other complications becomes available, the majority of pregnant individuals report that they would desire this information and would be more motivated to take recommended medications and to implement positive behavioral and educational changes in their pregnancy. If future prospective studies confirm that patients are more likely to adhere to recommendations with objective risk stratification, testing for preeclampsia risk could be an important tool driving behavior change to reduce the rate of preeclampsia.

### Supplementary Information

**Supplementary Material 1. **

**Supplementary Material 2. **

## Data Availability

The datasets used and/or analyzed during the current study are available from the corresponding author on reasonable request.
