# Supplementary material for: Impact of early preeclampsia prediction on medication adherence and behavior change: a survey of pregnant and recently-delivered individuals
Source: BMC Pregnancy Childbirth. 2024 Mar 13;24:196. doi: 10.1186/s12884-024-06397-z (PMC10935975; doi:10.1186/s12884-024-06397-z)
Supplement: Supplementary file 1 — Supplementary Material 1. [file 12884_2024_6397_MOESM1_ESM.docx]

| **Client Name:** | Mirvie | **Job Name:** | Pregnancy Study 2022 | |
| --- | --- | --- | --- | --- |
| **Wave No.**  **(srvyWave)** | N/A | **Job No. (dmJobNum)** | P148893 | |
| **Landing Page Title (scrIntroTitle)** | [We want your opinions!] | | | |
| **LOI for ISQ section (isqLOI)** | | | | [10] (minutes) |
| **Digital Fingerprinting and Fraud Score [Imperium RelevantID] (dfOptions)** By default, surveys will terminate any respondents who fail both of these tests and is recommended for panel sample. For client sample or vendor sample, the termination of DF or Fraud Score can be turned off if desired.  *[SELECT CODES 1-3]* | | | | 1 Digital Fingerprinting/Fraud Score  2 Terminate DF Duplicate  3 Terminate DF Fraud Score |
| **Mode of survey (srvyMode)** | | | | 1 - Web |
| **Sample Sources (list/vlist)**  List sample sources used for the study; Client, Dynata, Lucid, etc. | | | | MFOUR |
| **Respondent Type (srvyResp)**  Select ONE type of audience; if more than one select “Multiple” to indicate multiple audiences | | | | 1 – B2C |
| **Stagwell Company (srvyComp)**  v10.14 | | | | 1 – HIA (default) |
| **Other notes (i.e. Prior wave, programming notes, etc.)** | | | | None |

**SECTION 1: SAMPLE PRELOAD AND SCREENING QUESTIONS**

**BASE: ALL ONLINE RESPONDENTS (srvyMode/1)**

**scrIntroTitle** [INSERT LANDING PAGE TITLE]

**scrIntro**

During the survey, please do not use your browser's *FORWARD* and *BACK* buttons. Instead, please always use the button below to move through the survey. Please be aware that once you've answered a question, you might not be able to go back and change your answer.

The progress bar below indicates approximately what portion of the survey you have completed.

Simply click on the button at the bottom of the page to begin the survey.

**BASE:   ALL ONLINE RESPONDENTS (srvyMode/1)**

**clientConsent**

Welcome to our Survey.

Thank you for agreeing to take part in this research survey, which is conducted by The Harris Poll, a global consulting and market research firm, on behalf of a biotechnology company, working with several doctors and researchers.

The purpose of this research is to understand your healthcare experiences and to use that information to help people with similar experiences. This survey will take about 10 minutes to complete. You do not need to complete it all at one time.

You have the right to know the name of the sponsoring company. Therefore, at the end of this survey, we will reveal the name of the sponsoring company to you, to prevent potential biases in any responses.

The information you provide in the survey will be de-identified and The Harris Poll will not give anybody your name, contact information, or any information that can identify you. Participating in this survey in no way affects your healthcare, insurance, employment, or any other status since your personally identifiable information will not be shared.

Your participation would be greatly appreciated. If you have any questions, please contact The Harris Poll: [**healthcare_P148893@harrispoll.com**] or at 516-259-0012.

.

Please review The Harris Poll’s privacy policy for more information about how your data will be used and your choices.

Thank you.


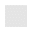


1.            I agree to continue

2.            I do not agree

[PN: IF ClientConsent/2 TERMINATE IMMEDIATELY. INSERT "privacy policy" AS HYPERLINK TO https://theharrispoll.com/privacy/]

**BASE: ALL RESPONDENTS**

**dmCntry** **[Country]** In which country or region do you currently reside?

[IF US STUDY, US LISTED FIRST ELSE CODES DISPLAYED IN ALPHABETICAL ORDER]

244. United States of America

14. Australia

33. Brazil

42. Canada

48. China

76. France

85. Germany

116. India

123. Italy

126. Japan

157. Mexico

196. Russian Federation [SUPPRESS]

215. Spain

243. United Kingdom

996. Other country

[PN: IF hCntry DOES NOT EQUAL dmCntry TERMINATE IMMEDIATELY.]

**BASE: ALL RESPONDENTS**

**dmAge [Age]** What is your age?

[RANGE 0 -120]

|_|_|_|

[PN: IF NOT 18-45 TERMINATE IMMEDIATELY.]

**BASE: ALL RESPONDENTS**

**dmStateUS [State (US)]** In what state or territory do you currently reside?

[DISPLAY IN DROP DOWN LIST]

1. Alabama

2. Alaska

3. Arizona

4. Arkansas

5. California

6. Colorado

7. Connecticut

8. Delaware

9. District of Columbia

10. Florida

11. Georgia

12. Hawaii

13. Idaho

14. Illinois

15. Indiana

16. Iowa

17. Kansas

18. Kentucky

19. Louisiana

20. Maine

21. Maryland

22. Massachusetts

23. Michigan

24. Minnesota

25. Mississippi

26. Missouri

27. Montana

28. Nebraska

29. Nevada

30. New Hampshire

31. New Jersey

32. New Mexico

33. New York

34. North Carolina

35. North Dakota

36. Ohio

37. Oklahoma

38. Oregon

39. Pennsylvania

40. Rhode Island

41. South Carolina

42. South Dakota

43. Tennessee

44. Texas

45. Utah

46. Vermont

47. Virginia

48. Washington

49. West Virginia

50. Wisconsin

51. Wyoming

52. American Samoa

53. Federated States of Micronesia

54. Guam

55. Marshall Islands

56. Northern Mariana Islands

57. Palau

58. Puerto Rico

59. Virgin Islands

[IF 18 AND FROM AL OR NE ((dmage/18) AND (dmstate/1,28)) )) OR IF 18-20 AND FROM MS (DMAGE/18-20 AND DMSTATE/25), CONTINUE THROUGH THE WHOLE SCREENER BUT THEN SEND TO DEMOS AND TERM]

**BASE: ALL US RESPONDENTS**

**dmZipUS [Zip Code (US)]** What is your zip code?

[5 DIGITS – DISPLAY ERROR IF ZIP CODE IS NOT VALID]

[IF dmZIPUS DOES NOT MATCH dmstate/US TERMIANTE IMMEDIATELY]

**BASE: ALL US RESPONDENTS**

**dmHispUS [Hispanic Origin (US)]** Are you of Hispanic, Latino, or Spanish origin?

1. Yes

2. No

[PROGRAMMER NOTE: INSERT “Why do we ask this question?” pop-up BELOW CHOICES]

**BASE: ALL US RESPONDENTS**

**dmRaceMUS [Race-Multi (US)]** What is your race? Please select all that apply.

[PROGRAMMER NOTE: DISPLAY IN ONE COLUMN.]

[MULTIPLE RESPONSE]

1. White

2. Black or African American

3. Native American or Alaskan Native

4. South Asian

5. Chinese

6. Korean

7. Japanese

8. Filipino

9. Arab/West Asian

12. Vietnamese

11. Other Asian

10. Native Hawaiian or Pacific Islander

13. Other race

[PROGRAMMER NOTE: INSERT “Why do we ask this question?” pop-up BELOW CHOICES]

**BASE: ALL RESPONDENTS**

**QS1** We are interested in learning about your experience with pregnancy.

  Which of the following describes you? Please select all that apply.

[MULTIPLE RESPONSE]

[PROGRAMMER NOTE: CHOICES 1 AND 2 MUST BE EXCLUSIVE OF EACH OTHER.]

[MAKE CODES 98 AND 99 EXCLUSIVE]

1. I am trying to get pregnant.

2. I am currently pregnant.

3. I gave birth in the last 12 months.

4. I gave birth in the last 13-24 months.

1. None of these
2. Decline to answer

 [IF QS1/NE2,3 CONTINUE THROUGH REST OF SCREENER BUT THEN SEND TO DEMOS THEN TERMINATE]

[DISPLAY QS2 AND QS3 ON ONE SCREEN]

**BASE:  PREGNANT (QS1/2)**

**Q2** What is your due date?  Please select the month and year below.  If you are not sure, please give your best estimate.

Month:   [DISPLAY IN DROP DOWN BOX]

1. January
2. February
3. March
4. April
5. May
6. June
7. July
8. August
9. September
10. October
11. November
12. December

**BASE: PREGNANT (QS1/2)**

**QS2b**

Year: [DISPLAY IN DROP DOWN BOX]

1. 2022
2. 2023

**BASE: ALL RESPONDENTS**

**QS3**How many times have you been pregnant in your lifetime? Please keep in mind that your responses will be kept confidential.

[DISPLAY IN DROP DOWN BOX]

1. 1 time
2. 2
3. 3
4. 4
5. 5
6. 6
7. 7
8. 8
9. 9
10. 10 or more times

97**. |_|** I have never been pregnant

99. |_| Decline to answer

**[IF QS3/97 SEND TO DEMOS THEN TERMINATE]**

**QUALIFICATIONS**

- Agrees to consent (ClientConsent/1)
- U.S. resident (dmCntry/244)
- Resident of Continental US (netRegionUS/1-4)
- Age 18-45 and not AL, MS, or NE ((dmage/18-45) AND (dmstateUS/NE 1, 25,28))
  - 19-45 in AL and NE ((dmage/19-45) AND (dmstateUS/1, 28))
  - 21-45 in MS (dmage/21-45) AND (dmstateUS/25)
- Currently pregnant (QS1/2) OR Has given birth within the last year (QS1/3)
- QS3/1-10,99

**QUOTAS**

- White respondents(net2RaceMUS/1) (n=400)
- Hispanic respondents(net2RaceMUS/2) (n=250)
- Black respondents(net2RaceMUS/3) (n=200)
- Asian respondents (net2RaceMUS/4) (n=100)
- All other respondents(net2RaceMUS/5) (n=50)

**SECTION 2: PERSONAL EXPERIENCE**

**BASE: NOT CURRENTLY PREGNANT (QS1/NE2)**

**Qintro.** You mentioned that you have given birth in the past 12 months. The following questions are focused on your pregnancy experiences, when answering, please think back to your previous pregnancy(ies).

**BASE: ALL QUALIFIED RESPONDENTS**

**Q200.** Overall, how satisfied are you with your pregnancy care from your healthcare provider(s)?

1. Very unsatisfied
2. Somewhat unsatisfied
3. Somewhat satisfied
4. Very satisfied

**BASE: ALL QUALIFIED RESPONDENTS**

**Q205.** How much do you disagree or agree with the following statements?

[COLUMNS; BANKED GRID]

1. Strongly disagree
2. Somewhat disagree
3. Somewhat agree
4. Strongly agree

[ROWS; RANDOMIZE; HOLD CODES 6-8 TOGETHER]

1. I would like my prenatal care to be personalized to my individual pregnancy. (For example, having a personalized care plan with education, visit schedules, monitoring, medications, lifestyle changes, and follow-up testing during pregnancy that fits my particular needs).
2. Overall, I feel my prenatal care has been personalized to meet my needs.
3. I trust the healthcare provider(s) who manage my pregnancy (such as an OB/GYN or midwife).
4. I feel heard when bringing up concerns about symptoms or concerns related to possible pregnancy complications with my healthcare provider(s).
5. I have felt at times like I don’t understand certain aspects of my pregnancy care as well as I would like to.
6. The more information I have about my pregnancy, the better the conversations are with my healthcare provider(s).
7. The more information I have about my pregnancy, the more empowered I feel.
8. The more information I have about my pregnancy, the more control I have over my care choices.
9. If there were a test that could predict my risk of complications in pregnancy, I would want it.

**SECTION 3: KNOWLEDGE OF PREECLAMPSIA**

**BASE: ALL QUALIFIED RESPONDENTS**

**Q300.** Now we are going to ask you a few questions about conditions that can affect people during pregnancy.

**BASE: ALL QUALIFIED RESPONDENTS**

**Q305.** How knowledgeable are you with the following terms?

[COLUMNS; BANKED GRID]

1. Never heard of
2. Not very knowledgeable
3. Somewhat knowledgeable
4. Very knowledgeable
5. Extremely knowledgeable

[ROWS; RANDOMIZE]

1. Preeclampsia
2. Development of diabetes in pregnancy
3. Down syndrome

**BASE: ALL QUALIFIED RESPONDENTS**

**Q310.**  Using the sliding scale below, in what percentage of pregnancies, on average, do the following occur? If you do not know, please use your best estimate.

[RANDOMIZE; SHOW SLIDER SCALE Between <1% and 15% starting at 0; ONLY SHOW STUBS 1,2,4 IF FAMILIAR AT Q305 (Q305C2-5/R1-3) ]

1. Development of preeclampsia
2. Development of diabetes in pregnancy
3. Premature birth
4. Down syndrome diagnosis

**BASE: THOSE WHO HAVE HEARD OF PREECLAMPSIA (Q305R1/C2-5)**

**Q315.** To the best of your knowledge, which of the following are warning signs of preeclampsia? Please select all that apply.

[RANDOMIZE; MULTIPLE RESPONSE; ANCHOR 96, 98; EXCLUSIVE 98]

1. Headache
2. Visual changes
3. Stomach pain (especially in the right upper side)
4. Swelling
5. Elevated blood pressure
6. Fatigue
7. Vaginal bleeding
8. Diarrhea
9. Contractions
10. Back pain

96. Other

1. Don’t know

**BASE: THOSE WHO HAVE HEARD OF PREECLAMPSIA (Q305R1/C2-5)**

**Q320.** How would you describe your risk of developing preeclampsia?

1. Below average
2. Average
3. Above average

98. Not at all sure

**BASE: THOSE WHO HAVE HEARD OF PREECLAMPSIA (Q305R1/C2-5)**

**Q325.** [Q320/1-3: You described your risk for developing preeclampsia as [INSERT RESPONSE FROM Q320 – lower case]. Why do you feel this way?]

[Q320/4: You mentioned you are not at all sure of your risk for developing preeclampsia. Why do you feel this way?]

[INSERT LARGE MANDATORY TEXT BOX]

**BASE: ALL QUALIFIED RESPONDENTS**

**Q330.** Have your healthcare provider(s) who are/who were managing your pregnancy discussed your personal risk of preeclampsia with you?

1. Yes
2. No

98. Do not recall

**BASE: ALL QUALIFIED RESPONDENTS**

**Q335.** How much do you disagree or agree with the following statements?

[COLUMNS; BANKED GRID]

1. Strongly disagree
2. Somewhat disagree
3. Somewhat agree
4. Strongly agree

[ROWS; RANDOMIZE]

1. I feel I have a good understanding of preeclampsia.
2. I feel I understand my personal risk of preeclampsia well.
3. I wish I knew more about my risk of preeclampsia and other pregnancy complications.
4. Before getting pregnant, I understood my risk of preeclampsia and other pregnancy complications.
5. I feel fully informed about my personal risk of complications during pregnancy, including preeclampsia.
6. I feel confident in my ability to get answers to my questions about preeclampsia and other pregnancy complications.
7. Outside of my healthcare provider(s), I know where to go to get reliable information about preeclampsia and other pregnancy complications.

**BASE: ALL QUALIFIED RESPONDENTS**

**Q340.** What complications, if any, have you ever experienced during any of your pregnancies?

[MANDATORY OPEN-END TEXT BOX]

1. Decline to answer

**BASE: ALL QUALIFIED RESPONDENTS**

**Q345.** Which complications have you experienced during any of your pregnancies? Please select all that apply.

[MULTIPLE RESPONSE; RANDOMIZE; ANCHOR AND EXCLUSIVE CODES 97 AND 99;]

1. Preeclampsia
2. Premature birth
3. Development of diabetes in pregnancy
4. None of these

99. Decline to answer

**SECTION 4: VALUE PREDICTION**

**BASE: ALL QUALIFIED RESPONDENTS**

**Q400** Now we are going to ask you some questions about testing during pregnancy. Before we do so, we have a brief paragraph for you to read.

**BASE: ALL QUALIFIED RESPONDENTS**

**Q401** Preeclampsia impacts ~8% of pregnancies, causing high blood pressure and other complications like kidney problems. If untreated or very severe, it can cause seizures and cardiovascular problems in the pregnant person and poor growth or stillbirth in babies. It often leads to premature birth, as the primary way to treat preeclampsia is delivery, and is a leading cause of injury and death during pregnancy.

There is currently no reliable way to predict preeclampsia. Pregnant patients and their doctors have to rely on generalized risk assessments based on pregnancy history, race, ethnicity, body mass index, and medical history which only predicts about 5% of cases, missing the majority.

With advance knowledge of which patients are predicted to develop preeclampsia, there are interventions, such as low dose aspirin and monitoring blood pressure, that can prevent or delay the onset of severe symptoms.

We would like to understand more about your perspective on having a prediction test for preeclampsia, and how this would impact you and your pregnancy care.

[PLEASE KEEP A TIMER HERE OF 30 SECONDS WITH THE CONTINUE BUTTON VISIBLE BUT GREYED. AFTER 30 SECONDS PARTICIPANT CAN PROCEED]

**BASE: ALL QUALIFIED RESPONDENTS**

**Q405** If a simple blood test predicted if you would get preeclampsia were available, what if any, of the following would you do or how would it make you feel? Please select all that apply.

[MULTIPLE RESPONSE; RANDOMIZE; ANCHOR CODES 96 AND 98; EXCLUSIVE CODE 98]

1. I would make different choices in the management of my pregnancy.
2. It would empower me to advocate for myself during my pregnancy.
3. It would help me feel more confident about notifying my care team with concerns about signs or symptoms.
4. It would help me feel more engaged with my pregnancy and care team.
5. I would want to set and follow a personalized treatment plan with my care team.
6. It would help strengthen my relationship with my care team.
7. It would give me some peace of mind.
8. It would add to my anxiety.

96. Other, please specify [MANDATORY TEXT BOX]

98. Don’t know

**BASE: ALL QUALIFIED RESPONDENTS**

**Q410.** How much do you disagree or agree with the following statements about screening and tests during pregnancy for preeclampsia?

[COLUMNS; BANKED GRID]

1. Strongly disagree
2. Somewhat disagree
3. Somewhat agree
4. Strongly agree

[ROWS; RANDOMIZE; ANCHOR 4 AFTER 3]t

1. Even if a prediction test were not 100% accurate, I would want to take a test early in my pregnancy that lets me know my chances of developing a problem like preeclampsia.
2. If a prediction test showed my risk to develop preeclampsia was low, I would feel more at ease about my prenatal care.
3. If I better understood the risks of preeclampsia, I would be more motivated to follow my healthcare provider(s)’ medication recommendations.
4. If I better understood the risks of preeclampsia, I would be more motivated to follow my healthcare provider(s)’ recommendation to take baby aspirin.
5. If a screening test told me I was at higher risk for preeclampsia, I would be interested in options to monitor my blood pressure at home.
6. If a screening test told me I was at higher risk for preeclampsia, I would expect that my healthcare provider(s) would make a personalized plan for my pregnancy care.
7. If a screening test told me I was at higher risk for preeclampsia, I would want to discuss the signs and symptoms of preeclampsia with my healthcare provider(s) (such as an OB/GYN or midwife) to make sure I know when to call them.

**BASE: ALL QUALIFIED RESPONDENTS**

**Q415.** How likely would you be to take medications, such as aspirin, throughout your pregnancy if your healthcare provider(s)recommended it?

1. Very unlikely
2. Somewhat unlikely
3. Somewhat likely
4. Very likely

**BASE: ALL QUALIFIED RESPONDENTS**

**Q420.** You mentioned you would be [INSERT RESEPONSE FROM Q415 – lower case] to take medications, such as aspirin, throughout your pregnancy if your healthcare provider(s) recommended it.

If, when making this recommendation, they told you it was because your risk of developing preeclampsia was high, would this increase, decrease, or have no change on your likelihood to take the medication?

- - - 1. Significantly decrease
      2. Slightly decrease
      3. No change
      4. Slightly increase
      5. Significantly increase

**BASE: ALL QUALIFIED RESPONDENTS**

**Q425.** You mentioned you would be [INSERT RESEPONSE FROM Q415 – lower case] to take medications, such as aspirin, throughout your pregnancy if your healthcare provider(s) recommended it.

If, when making this recommendation, they told you your high risk for developing preeclampsia was determined using a prediction test, would this increase, decrease, or have no change on your likelihood to take the medication?

- - - 1. Significantly decrease
      2. Slightly decrease
      3. No change
      4. Slightly increase
      5. Significantly increase

**WEBOGRAPHICS**

**BASE: ALL US RESPONDENTS AGE 18+**

**web9306** Have you chosen not to purchase a product or service, or told someone else not to purchase a product or service, made by a particular company because the company’s actions or policies conflicted with your values or beliefs?

1. Yes, in the past year

2. Yes, more than 1 year ago but within the past 2 years

3. Yes, more than 2 years ago but within the past 3 years

4. Yes, more than 3 years ago

5. No, I have never done this

**BASE: US RESPONDENTS AGE 18+**

**web9314** Most companies today want to know about the individual interests and lifestyle of their customers so they can tailor their information services and products to each customer’s personal preferences. In general, do you see such personalization as a good thing?

1. Yes

2. No

**BASE: US RESPONDENTS AGE 18+**

**web9318** On how many different occasions did you do vigorous physical exercise during the past 30 days?

RANGE: 0-120

NUMERIC BOX

l__ll__l__l

**BASE: US RESPONDENTS AGE 18+**

**web9334** On how many separate occasions would you say you watched news programs on TV during the past 30 days?

RANGE: 0-120

NUMERIC BOX

l__ll__l__l

**BASE: US RESPONDENTS AGE 18+**

**web9336** Do you think that community service should be a requirement in schools?

1. Yes

2. No

**SECTION 5: DEMOGRAPHICS**

**BASE: ALL RESPONDENTS**

**dmGenM [Gender-Multi]** Are you…? Please select all that apply.

[PN: MULTIPLE RESPONSE – CODES 1 & 2 CANNOT BE SELECTED TOGETHER – EXCLUSIVE 9]

1. Male

2. Female

3. Transgender

4. Non-binary or Gender Non-conforming

9. Prefer not to answer]

BASE: ALL RESPONDENTS

finGen HIDDEN: Gender (Final) - USE FOR WEIGHTING ONLY, NOT FOR DELIVERABLES

[IF dmGen/dmGenM=1, SELECT 1; IF dmGen/dmGenM=2, SELECT 2; IF dmGen/dmGenM<>1 OR 2 THEN RANDOMIZE 50/50]

1. Male

2. Female

**BASE: ALL US RESPONDENTS AND 18+**

**dmEduUS [Education (US)]**  What is the highest level of education you have completed?

[SINGLE RESPONSE]

[PROGRAMMER NOTE: DISPLAY IN ONE COLUMN, GOING DOWN.]

1. Less than high school

2. Completed some high school

3. High school graduate

4. Job-specific training program(s) after high school

5. Some college, but no degree

6. Associate degree

7. Bachelor’s degree (such as B.A., B.S.)

8. Some graduate school, but no degree

9. Graduate degree (such as MBA, MS, M.D., Ph.D.)

**BASE: ALL US RESPONDENTS 18+**

**dmHhIncUS [Household Income (US)]**  How much total combined income did all members of your household earn before taxes last year?

*This includes money from jobs; net income from business, farm, or rent; pensions; dividends; interest; social security payments; and any other money income received by members of your household who are eighteen (18) years of age or older.*

[PROGRAMMER NOTE: DISPLAY IN ONE COLUMN, GOING DOWN.]

1. Less than $15,000

2. $15,000 to $24,999

3. $25,000 to $34,999

4. $35,000 to $49,999

5. $50,000 to $74,999

6. $75,000 to $99,999

7. $100,000 to $124,999

8. $125,000 to $149,999

9. $150,000 to $199,999

10. $200,000 to $249,999

11. $250,000 or more

[PROGRAMMER NOTE: INSERT “Why do we ask this question?” pop-up BELOW CHOICES]

**BASE: ALL RESPONDENTS**

**dmNetUse [Internet Usage]** Please indicate how much time you spend on the Internet in an average week. Please include all places you might log on, such as at home/work/elsewhere by desktop, laptop, tablet, smartphone, etc.

1. Less than 1 hour

2. 1 - 4 hours

3. 5 - 9 hours

4. 10 - 19 hours

5. 20 hours or more

**BASE: ALL RESPONDENTS AND 18+**

**dmMarStat [Marital Status]** What is your current marital status?

1. Never married

2. Married or civil union

3. Divorced

4. Separated

5. Widowed

6. Living with partner

**BASE: ALL RESPONDENTS**

**dmAdultHh [Adults in Household]**

[IF AGE 18 OR OVER (dmAge/>17)]

Including yourself, how many people age 18 or older live in your household?

[IF AGE 17 AND YOUNGER (dmAge/<18)]

How many people age 18 or older live in your household? If you live in more than one household, please answer for only one of the households.

[IF AGE (dmAge/>17) RANGE: 1-50]

[IF AGE (dmAge/<18) RANGE: 0-50]

|_|_|

**BASE: ALL RESPONDENTS**

**dmChildHh [Children in Household]**

[IF AGE 18 OR OVER (dmAge/>17)]

How many people under the age of 18 live in your household?

[IF AGE 17 OR YOUNGER (dmAge/<18)]

Including yourself, how many people under the age of 18 live in your household? If you live in more than one household, please answer for only one of the households.

[IF AGE (dmAge/>17) RANGE: 0-15]

[IF AGE (dmAge/<18) RANGE: 1-15]

|_|_|

**BASE: ALL RESPONDENTS 18+**

**dmEmploy [Employment Status]** Which of the following best describes your employment status?

[SINGLE RESPONSE]

1. Employed full time

2. Employed part time

3. Self-employed full time

4. Self-employed part time

5. Not employed, but looking for work

6. Not employed and not looking for work

7. Not employed, unable to work due to a disability or illness

8. Retired

9. Student

10. Stay-at-home spouse or partner

**BASE: ALL RESPONDENTS**

**clientEnd** As mentioned at the beginning of the survey we can now reveal that this research is sponsored by Mirvie.
