# Supplementary material for: Impact of early preeclampsia prediction on medication adherence and behavior change: a survey of pregnant and recently-delivered individuals
Source: BMC Pregnancy Childbirth. 2024 Mar 13;24:196. doi: 10.1186/s12884-024-06397-z (PMC10935975; doi:10.1186/s12884-024-06397-z)
Supplement: Supplementary file 2 — Supplementary Material 2. [file 12884_2024_6397_MOESM2_ESM.pdf]

Supplemental Tables *:

* - Benjamini-Hochberg adjusted p-values (fdr's) of the corresponding pairwise proportion tests are reported in the columns following the "total" column

Supplemental Table 1: Satisfaction with Care by Race

Race	Unsatisfied	Satisfied	total	Asian	Black	Hispanic
Asian	8 (15.1%)	45 (84.9%)	53			
Black	32 (20%)	128 (80%)	160	0.56		
Hispanic	41 (15.2%)	229 (84.8%)	270	1.00	0.25	
White	77 (15%)	438 (85%)	515	1.00	0.16	1

Supplemental Table 2: Participant sentiments on pregnancy care, personalized care, and feeling heard by race.

(Cronbach's alpha = 0.76)

Question	Race	Disagree	Agree	total	Asian	Black	Hispanic
The more information I have about my pregnancy, the more control I have over my care choices.	Asian	2 (3.8%)	51 (96.2%)	53			
	Black	8 (5%)	152 (95%)	160	1.0000		
	Hispanic	9 (3.3%)	261 (96.7%)	270	1.0000	1.0000	

	White	18 (3.5%)	497 (96.5%)	515	1.0000	1.0000	1.0000
The more information I have about my pregnancy, the better the conversations with my provider (s).	Asian	1 (1.9%)	52 (98.1%)	53			
	Black	4 (2.5%)	156 (97.5%)	160	1.0000		
	Hispanic	16 (5.9%)	254 (94.1%)	270	0.6697	0.6697	

	White	24 (4.7%)	491 (95.3%)	515	0.6697	0.6697	0.6697
The more information I have about my pregnancy, the more empowered I feel.	Asian	2 (3.8%)	51 (96.2%)	53			
	Black	11 (6.9%)	149 (93.1%)	160	0.7520		
	Hispanic	28 (10.4%)	242 (89.6%)	270	0.4197	0.4432	

	White	18 (3.5%)	497 (96.5%)	515	1.0000	0.3167	0.0011
I would like my prenatal care to be personalized to my individual pregnancy.	Asian	5 (9.4%)	48 (90.6%)	53			
	Black	8 (5%)	152 (95%)	160	0.8058		
	Hispanic	18 (6.7%)	252 (93.3%)	270	0.8058	0.8058	

	White	26 (5%)	489 (95%)	515	0.8058	1.0000	0.8058
I trust the healthcare provider(s) who manage my pregnancy (such as an OB/GYN or midwife.)	Asian	4 (7.5%)	49 (92.5%)	53			
	Black	20 (12.5%)	140 (87.5%)	160	0.7801		
	Hispanic	27 (10%)	243 (90%)	270	0.9177	0.7801	

	White	33 (6.4%)	482 (93.6%)	515	0.9778	0.1175	0.2919
If there were a test that could predict my risk of complications in pregnancy, I would want it.	Asian	4 (7.5%)	49 (92.5%)	53			
	Black	14 (8.8%)	146 (91.2%)	160	1.0000		
	Hispanic	15 (5.6%)	255 (94.4%)	270	0.9685	0.8282	

	White	58 (11.3%)	457 (88.7%)	515	0.8282	0.8282	0.0776
Overall, I feel my prenatal care has been personalized to meet my needs.	Asian	8 (15.1%)	45 (84.9%)	53			
	Black	28 (17.5%)	132 (82.5%)	160	1.0000		
	Hispanic	39 (14.4%)	231 (85.6%)	270	1.0000	0.7194	
	White	55 (10.7%)	460 (89.3%)	515	0.7194	0.1861	0.4602

I feel heard when bringing up concerns about symptoms or concerns related to possible pregnancy complications with my healthcare provider(s).	Asian	7 (13.2%)	46 (86.8%)	53	
	Black	34 (21.2%)	126 (78.8%)	160	0.5548

	Hispanic	37 (13.7%)	233 (86.3%)	270	1.0000	0.1712	
	White	65 (12.6%)	450 (87.4%)	515	1.0000	0.0616	1.0000
I have felt at times like I don’t understand certain aspects of my pregnancy care as well as I would like to.	Asian	19 (35.8%)	34 (64.2%)	53			

Black	57 (35.6%)	103 (64.4%)	160	1.0000		
Hispanic	110 (40.7%)	160 (59.3%)	270	0.7309	0.5134	
White	231 (44.9%)	284 (55.1%)	515	0.5134	0.2929	0.5134

Question	Race	Disagree	Agree	total	Asian	Black	Hispanic

If a screening test told me I was at higher risk for preeclampsia, I would want to discuss the signs and symptoms of preeclampsia with my healthcare provider(s) to make sure I know when to call them.	Asian	1 (1.9%)	52 (98.1%)	53			
	Black	4 (2.5%)	156 (97.5%)	160	1.0000		
	Hispanic	22 (8.1%)	248 (91.9%)	270	0.3681	0.1819	
	White	25 (4.9%)	490 (95.1%)	515	0.6272	0.4339	0.2734

If a screening test told me I was at higher risk for preeclampsia, I would expect that my healthcare provider(s) would make a personalized plan for my pregnancy care.	Asian	2 (3.8%)	51 (96.2%)	53			
	Black	12 (7.5%)	148 (92.5%)	160	0.7940		
	Hispanic	21 (7.8%)	249 (92.2%)	270	0.7940	1.0000	
	White	21 (4.1%)	494 (95.9%)	515	1.0000	0.3680	0.2594

If a screening test told me I was at higher risk for preeclampsia, I would be interested in options to monitor my blood pressure at home.	Asian	1 (1.9%)	52 (98.1%)	53			
	Black	11 (6.9%)	149 (93.1%)	160	0.8041		
	Hispanic	17 (6.3%)	253 (93.7%)	270	0.8041	0.9737	
	White	29 (5.6%)	486 (94.4%)	515	0.8041	0.9737	0.9737
If a prediction test showed my risk to develop preeclampsia was low, I would feel	Asian	1 (1.9%)	52 (98.1%)	53			

more at ease about my prenatal care.							
	Black	14 (8.8%)	146 (91.2%)	160	0.3334		
	Hispanic	25 (9.3%)	245 (90.7%)	270	0.3334	1.0000	
	White	44 (8.5%)	471 (91.5%)	515	0.3334	1.0000	1.0000
Even if a prediction test were not 100% accurate, I would want to take a test early in my pregnancy that lets me know my chances of developing a	Asian	3 (5.7%)	50 (94.3%)	53			

problem like preeclampsia.							
	Black	15 (9.4%)	145 (90.6%)	160	0.7190		
	Hispanic	15 (5.6%)	255 (94.4%)	270	1.0000	0.5737	
	White	58 (11.3%)	457 (88.7%)	515	0.6143	0.7190	0.0776
If I better understood the risks of preeclampsia, I would be more motivated to follow my healthcare	Asian	4 (7.5%)	49 (92.5%)	53			

provider(s) medication recommendations.							
	Black	18 (11.2%)	142 (88.8%)	160	0.7343		
	Hispanic	32 (11.9%)	238 (88.1%)	270	0.7343	0.9740	
	White	70 (13.6%)	445 (86.4%)	515	0.7343	0.7343	0.7343
If I better understood the risks of preeclampsia, I would be more	Asian	3 (5.7%)	50 (94.3%)	53			

motivated to follow my healthcare provider(s) recommendation to take baby aspirin.							
	Black	17 (10.6%)	143 (89.4%)	160	0.5069		
	Hispanic	30 (11.1%)	240 (88.9%)	270	0.5069	1.0000	
	White	77 (15%)	438 (85%)	515	0.4226	0.4226	0.4226

Supplemental Table 3: Blood test for preeclampsia prediction: impact on care and self-reported motivation to act on results by race and ethnicity (Cronbach's alpha = 0.86)

Question	Race	Disagree	Agree	total	Asian	Black	Hispanic

If a screening test told me I was at higher risk for preeclampsia, I would want to discuss the signs and symptoms of preeclampsia with my healthcare provider(s) to make sure I know when to call them.	Asian	1 (1.9%)	52 (98.1%)	53			
	Black	4 (2.5%)	156 (97.5%)	160	1.0000		
	Hispanic	22 (8.1%)	248 (91.9%)	270	0.3681	0.1819	
	White	25 (4.9%)	490 (95.1%)	515	0.6272	0.4339	0.2734
If a screening test told me I was at higher risk for preeclampsia, I would expect that my healthcare provider(s) would make a personalized plan for my pregnancy care.	Asian	2 (3.8%)	51 (96.2%)	53			
	Black	12 (7.5%)	148 (92.5%)	160	0.7940		

	Hispanic	21 (7.8%)	249 (92.2%)	270	0.7940	1.0000	
	White	21 (4.1%)	494 (95.9%)	515	1.0000	0.3680	0.2594
If a screening test told me I was at higher risk for preeclampsia, I would be interested in options to monitor my blood pressure at home.	Asian	1 (1.9%)	52 (98.1%)	53			
	Black	11 (6.9%)	149 (93.1%)	160	0.8041		
	Hispanic	17 (6.3%)	253 (93.7%)	270	0.8041	0.9737	
	White	29 (5.6%)	486 (94.4%)	515	0.8041	0.9737	0.9737
If a prediction test showed my risk to develop preeclampsia was low, I would feel more at ease about my prenatal care.	Asian	1 (1.9%)	52 (98.1%)	53			
	Black	14 (8.8%)	146 (91.2%)	160	0.3334		

	Hispanic	25 (9.3%)	245 (90.7%)	270	0.3334	1.0000	
	White	44 (8.5%)	471 (91.5%)	515	0.3334	1.0000	1.0000
Even if a prediction test were not 100% accurate, I would want to take a test early in my pregnancy that lets me know my chances of developing a problem like preeclampsia.	Asian	3 (5.7%)	50 (94.3%)	53			
	Black	15 (9.4%)	145 (90.6%)	160	0.7190		
	Hispanic	15 (5.6%)	255 (94.4%)	270	1.0000	0.5737	
	White	58 (11.3%)	457 (88.7%)	515	0.6143	0.7190	0.0776
If I better understood the risks of preeclampsia, I would be more motivated to follow my healthcare provider(s) medication recommendations.	Asian	4 (7.5%)	49 (92.5%)	53			

	Black	18 (11.2%)	142 (88.8%)	160	0.7343		
	Hispanic	32 (11.9%)	238 (88.1%)	270	0.7343	0.9740	
	White	70 (13.6%)	445 (86.4%)	515	0.7343	0.7343	0.7343
If I better understood the risks of preeclampsia, I would be more motivated to follow my healthcare provider(s) recommendation to take baby aspirin.	Asian	3 (5.7%)	50 (94.3%)	53			
	Black	17 (10.6%)	143 (89.4%)	160	0.5069		
	Hispanic	30 (11.1%)	240 (88.9%)	270	0.5069	1.0000	
	White	77 (15%)	438 (85%)	515	0.4226	0.4226	0.4226
